# Bile acid effects are mediated by ATP release and purinergic signalling in exocrine pancreatic cells

**DOI:** 10.1186/s12964-015-0107-9

**Published:** 2015-06-09

**Authors:** Justyna M. Kowal, Kristian A. Haanes, Nynne M. Christensen, Ivana Novak

**Affiliations:** Department of Biology, Section for Cell Biology and Physiology, August Krogh Building, University of Copenhagen, Universitetsparken 13, DK-2100 Copenhagen, Denmark; Present address: Department of Clinical Experimental Research, Glostrup Research Institute, Copenhagen University Hospital, Glostrup, Denmark

**Keywords:** ATP release, P2 receptors, Bile acids, CDCA, TGR5, FXR, Ca^2+^, Pancreas, ATP sensor, AT1.03^YEMK^, FLIM-FRET

## Abstract

**Background:**

In many cells, bile acids (BAs) have a multitude of effects, some of which may be mediated by specific receptors such the TGR5 or FXR receptors. In pancreas systemic BAs, as well as intra-ductal BAs from bile reflux, can affect pancreatic secretion. Extracellular ATP and purinergic signalling are other important regulators of similar secretory mechanisms in pancreas. The aim of our study was to elucidate whether there is interplay between ATP and BA signalling.

**Results:**

Here we show that CDCA (chenodeoxycholic acid) caused fast and concentration-dependent ATP release from acini (AR42J) and duct cells (Capan-1). Taurine and glycine conjugated forms of CDCA had smaller effects on ATP release in Capan-1 cells. In duct monolayers, CDCA stimulated ATP release mainly from the luminal membrane; the releasing mechanisms involved both vesicular and non-vesicular secretion pathways. Duct cells were not depleted of intracellular ATP with CDCA, but acinar cells lost some ATP, as detected by several methods including ATP sensor AT1.03^YEMK^. In duct cells, CDCA caused reversible increase in the intracellular Ca^2+^ concentration [Ca^2 +^]_i_, which could be significantly inhibited by antagonists of purinergic receptors. The TGR5 receptor, expressed on the luminal side of pancreatic ducts, was not involved in ATP release and Ca^2+^ signals, but could stimulate Na^+^/Ca^2+^ exchange in some conditions.

**Conclusions:**

CDCA evokes significant ATP release that can stimulate purinergic receptors, which in turn increase [Ca^2+^]_i_. The TGR5 receptor is not involved in these processes but can play a protective role at high intracellular Ca^2+^ conditions. We propose that purinergic signalling could be taken into consideration in other cells/organs, and thereby potentially explain some of the multifaceted effects of BAs.

**Electronic supplementary material:**

The online version of this article (doi:10.1186/s12964-015-0107-9) contains supplementary material, which is available to authorized users.

## Lay abstract

In recent years there has been a growing interest for the role of bile acids as signalling molecules in many cells/organs. Several types of bile acids receptors have been identified, but some modulatory functions of bile acids remain unexplained. Here, we show that bile acids, in particular chenodeoxycholic acid, causes significant release of ATP from exocrine pancreatic cells. Extracellular ATP can then via purinergic receptors regulate or co-regulate epithelial functions, such as pancreatic duct secretion, which is important for normal digestive processes. Our study brings novel insights into regulation of pancreas functions. Moreover, we propose that purinergic signalling should be taken into consideration in other cells/organ types, as it could potentially explain some of the multifaceted effects of bile acids.

## Background

Bile acids (BAs) are natural amphiphilic metabolites originating from cholesterol degradation. The main human primary bile acids are chenodeoxycholic acid (CDCA) and cholic acid (CA), which can be conjugated with glycine and taurine to form bile salts. The role of bile acids as signalling molecules and as targets for drug development gained interest during the last years, and several bile acid receptors were discovered, including the nuclear farnesoid receptor (FXR) and the membrane receptor TGR5 (GPBAR1) [1–4]. TGR5 is expressed in many cell types and regulates a variety of functions. For example, TGR5 modulates liver function, glucose metabolism and insulin sensitivity, and immune responses [4–7]. In several epithelia such as colon, respiratory epithelia and bile ducts, BAs regulate ion transport, at least partly via TGR5 or FXR receptors [8–10]. For example, in bile ducts, TGR5 stimulates biliary HCO_3_^−^ and fluid secretion [11–14].

In pancreas, BAs have multiple effects. In the endocrine pancreas, tauroursodeoxycholate has a protective role on pancreatic islets as it decreases apoptosis and stimulates insulin secretion after stress conditions [15]. It is reported that BAs can stimulate both FXR and TGR5 receptors in mouse β-cells and induce fast insulin secretion [16, 17]. Furthermore, there is TGR5-dependent stimulation of glucagon-like-peptide-1 (GLP-1) release from enterocytes [18], and then systemic GLP-1 increases insulin secretion from β-cells [19]. In exocrine pancreas, BAs can exert effects on several levels, as they can reach pancreas not only systemically, but also via reflux of bile into pancreatic ducts. Pancreatic acini express BA transporters [20] and some BAs at low concentrations can activate Ca^2+^-independent cation currents [21]. At high concentrations BAs can evoke cytotoxicaly high intracellular Ca^2+^ concentrations, [Ca^2+^]_i_, in pancreatic acini. This is due to inhibition of the sacro/endoplasmatic reticulum Ca^2+^-ATPases (SERCA), release of Ca^2+^ from ER and acidic stores and granules, increased Ca^2+^ influx, and acini show cellular acidosis, enzyme activation and mitochondrial malfunction, which can eventually lead to development of (biliary) acute pancreatitis [20, 22–24]. In one study, TGR5/GPBAR1 receptor knockout mice had less severe pancreatitis after infusion of BAs [25]. For pancreatic ducts, it has been proposed that BAs can stimulate duct secretion and they can tolerate higher BA concentrations [26–28]. Several studies show that BAs stimulate Cl^−^ and K^+^ channels; where the later have been identified as large conductance Ca^2+^-activated K^+^ channels (BK, K_Ca_1.1), but identity of Cl^−^ channels is not clear [26, 29, 30].

Another important regulatory system in pancreatic ducts is the purinergic signalling. Extracellular ATP can, via a number of P2 receptors that stimulate Ca^2+^ signalling, regulate Cl^−^ and K^+^ channels and acid/base transporters and thereby modulate HCO_3_^−^ and fluid secretion [31, 32]. Until now it has not been investigated whether pancreatic ducts release ATP, however, it is well established that ATP is released from pancreatic acini, which store ATP in zymogen granules, where it is accumulated by the Vesicular NUcleotide Transporter (VNUT, *SLC17A9*) [33]. Acinar ATP is released by exocytosis into the duct lumen in response to cholinergic or hormonal stimulation [34, 35]. In various other cells, ATP release can occur also via ion channels/transporters, such as maxi-anion channels, connexins, pannexins with/without P2X7 receptors [36, 37].

Considering that BAs and purinergic signalling appear to have similar paracrine effects on ion and fluid transport in exocrine pancreas, we hypothesized whether there was any interaction between these two intra-ductal regulatory systems. Therefore, we designed a study to test whether BAs, i.e., chenodeoxycholic acid (CDCA) and its glycine and taurine conjugated forms (GCDCA and TCDCA) can affect ATP release, and whether CDCA also influence intracellular ATP levels. A further aim was to elucidate whether BA signalling involves TGR5 or FXR receptors in the ATP release process and/or P2 receptors and downstream Ca^2+^ signalling. For this purpose we used duct and acini models (Capan-1 and AR42J cells), live cell luminescence assay for extracellular ATP, intracellular ATP sensors, AT1.03^YEMK^, and [Ca^2+^]_i._ imaging. Our study shows that CDCA indeed caused a very fast release of ATP from exocrine pancreatic cells via both non-exocytotic and vesicular pathways. Furthermore, a significant part of the CDCA effect on Ca^2+^ signalling is mediated via purinergic signalling. We also demonstrate the presence of the TGR5 and FXR receptors in human pancreatic ducts and show that TGR5 receptor can prevent intracellular Ca^2+^ overloads possibly by stimulation of the Na^+^/Ca^2+^ exchanger.

## Results

### CDCA but not GCDCA and TCDCA stimulates high ATP release from pancreatic cells

**Fig. 1:**
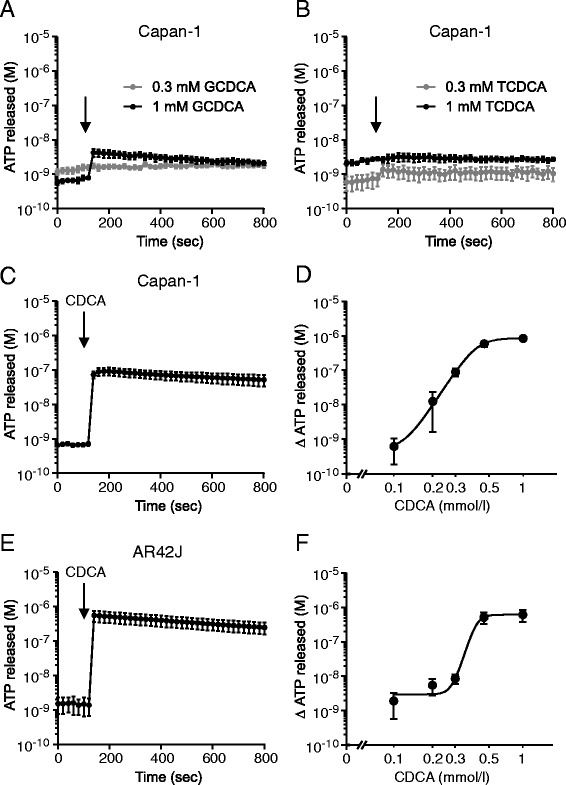
CDCA induced ATP release from exocrine pancreatic cells. The time-course of ATP release from Capan-1 cells in response to 0.3 and 1 mM of (**a**) GCDCA and (**b**) TCDCA (n = 4, 4). The time-course of ATP release from (**c**) Capan-1 and (**e**) AR42J after stimulation with 0.3 and 0.5 mM CDCA, respectively (n = 6, 5). Dose-dependent release of ATP from (**d**) Capan-1 and (**f**) AR42J cells in response to CDCA (n = 5, 6). The EC_50_ values of ATP release from AR42J and Capan-1 are 0.44 mM (pEC_50_ = 3.36 ± 0.03) and 0.43 mM (pEC_50_ 3.36 ± 0.04) (n = 5, 6) respectively in the range of 0.1 – 1 mM CDCA. Y-axis shows ATP concentrations, which were corrected for 10^6^ cells per 1 ml (see Methods). Data shown as mean values ± SEM. Arrows indicate addition of stimulants

ATP release on the whole organ level is difficult to detect due to action of membrane-bound and soluble nucleotidases [35, 38]. Therefore, ATP release and autocrine/paracrine signalling is usually studied on isolated cells or cell lines. In the first series of experiments we investigated the effect of several bile acids: GCDCA (glycochenodeoxycholic acid); TCDCA (taurochenodeoxycholic acid); and CDCA (chenodeoxycholic acid) on ATP release from Capan-1 cells, which are a model of pancreatic ducts. Time resolved luminescence recordings revealed that 0.3 mM of GCDCA and TCDCA had minor effects on ATP release from these cells (Fig. 1 a-b). At higher concentrations (1 mM) GCDCA evoked significant but small increase in extracellular ATP, ATP_e_, by 4.2 ± 0.8 nM (n = 4) above the basal level. TCDCA (1 mM) had no effect on ATP release. In contrast to GCDCA and TCDCA, CDCA (0.3 mM) caused a fast and substantial ATP release in Capan-1 cells (Fig. 1c). Therefore, we investigated the effect of CDCA concentrations on ATP release from Capan-1 (Fig. 1d). Furthermore, we investigated the effect of CDCA on AR42J cells, which are a model for pancreatic acinar cells (Fig. 1 e, f). Fig. 1 c-f shows that the duct and acinar cells released ATP in a concentration-dependent manner in a narrow range from 0.1 to 1 mM CDCA, and EC_50_ values are 0.43 mM and 0.44 mM for Capan-1 and AR42J cells, respectively. The peak/maximum ATP release observed after stimulation with 1 mM CDCA was 848 ± 16 nM (n = 6) for Capan-1 and 614 ± 79 nM (n = 5) for AR42J cells. These data show that CDCA can increase extracellular ATP concentrations by a factor of 100–1000 above the baseline. In all the following experiments, Capan-1 cells were stimulated with 0.3 mM CDCA and AR42J cells with 0.5 mM CDCA.

**Fig. 2:**
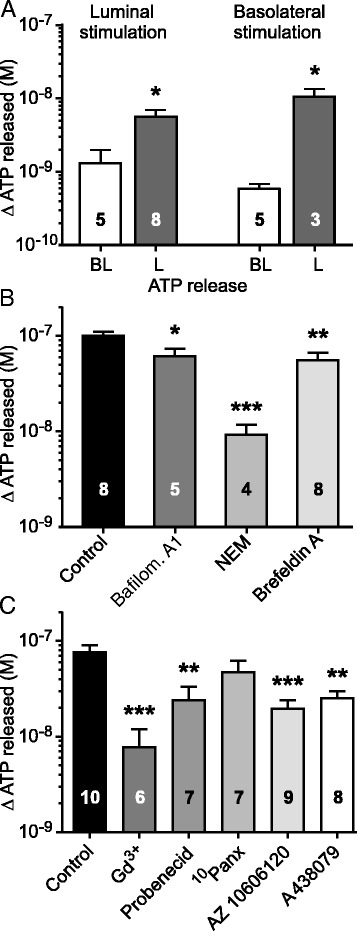
Sidedness of ATP release and the effect of vesicular and non-vesicular inhibitors on ATP release pathways. **a** Mean values of ATP released across basolateral (BL) and luminal (L) sides from Capan-1 cells after 1 min of apical or serosal stimulation with 0.3 mM CDCA. **b** Inhibitors of vesicular transport decreased ATP release from Capan-1 cells in response to 0.3 mM CDCA. Cells were incubated with vacuolar-type H^+^-ATPase inhibitor (bafilomycin A1, 1 μM; n = 5), vesicle fusion inhibitor N-Ethylmaleimide (NEM, 250 μM, n = 4) and brefeldin A (5 μg/ml; n = 8). **c** Influence of the non-vesicular transport inhibitors: gadolinium chloride (Gd^3+^, 50 μM; n = 6), probenecid (500 μM; n = 7), ^10^Panx - mimetic pannexin peptide (100 μM; n = 7), as well as P2X7 receptor inhibitors AZ10606120 (10 μM; n = 9) and A438079 (10 μM; n = 7) on CDCA-induced ATP release from Capan-1 cells is shown. Results are given as mean net values ± SEM. *= *P* < 0.05, **= *P* < 0.01, ***= *P* < 0.001

Since Capan-1 cells are a model of human pancreatic duct epithelium, it was relevant to investigate whether ATP release is polarized, i.e., whether it occurs preferentially across the luminal or the basolateral membrane. Capan-1 cells were cultured as polarized monolayers, and CDCA was administered luminally or basolaterally. After the luminal stimulation with 0.3 mM CDCA (Fig. 2a), there was significantly higher ATP release from the luminal side (5.7 ± 1.3 nM, n = 8) compared to the basolateral side (1.3 ± 0.7 nM, n = 5). Interestingly, a similar high luminal ATP release was observed when monolayers were stimulated with basolateral CDCA (Fig. 2a). ATP released across the luminal side was 10.5 ± 2.9 nM (n = 3) compared to the basolateral side 0.6 ± 0.03 nM (n = 5). Notably, these ATP values made in offline analysis of samples were lower than in online measurements, most likely due to different sampling volumes and ATP hydrolysis by ecto-nucleotidases [39, 40].

### CDCA induces ATP release via multiple pathways

In order to identify the pathways involved in CDCA-induced ATP release, Capan-1 cells were incubated with vesicular and non-vesicular transport inhibitors. Fig. 2b shows that CDCA caused high ATP release of 100 ± 10 nM (n = 8), and after incubation with bafilomycin A1, an inhibitor for vacuolar-type H^+^-ATPase, ATP release was significantly reduced to 61 ± 12 nM (n = 5). Inhibition of CDCA-evoked vesicular ATP release was also observed in the presence of N-Ethylmaleimide, an inhibitor of vesicle fusion, which markedly supressed ATP release to 9 ± 3 nM (n = 4). In addition, reducing vesicular transport from ER to Golgi by brefeldin A also decreased the ATP release to 55 ± 11 nM (n = 8). In another series of experiments, we tested the effect of non-vesicular transport inhibitors (Fig. 2c). Data presented in Fig. 2c, show that Gd^3+^, which inhibits pannexin and connexin hemichannels in addition to maxi anion channels, markedly inhibited CDCA-induced ATP release to 8 ± 4 nM (n = 6) compared to control of 76 ± 15 nM (n = 10). When cells were treated with probenecid, there was a significant decrease in ATP release to 24 ± 9 nM (n = 7). The pannexin-1 inhibitor, ^10^Panx, tended to decrease ATP release to 47 ± 15 nM (n = 7), but no statistical significance was reached. Two P2X7 receptor antagonists significantly inhibited the ATP release to 19 ± 5 nM (AZ10606120, n = 9) and 25 ± 5 nM (A438079, n = 8). Taken together, present data indicate that both vesicular and non-vesicular mechanisms are involved in CDCA-induced ATP release from pancreatic epithelial cells.

### Effect of CDCA on intracellular ATP

**Fig. 3:**
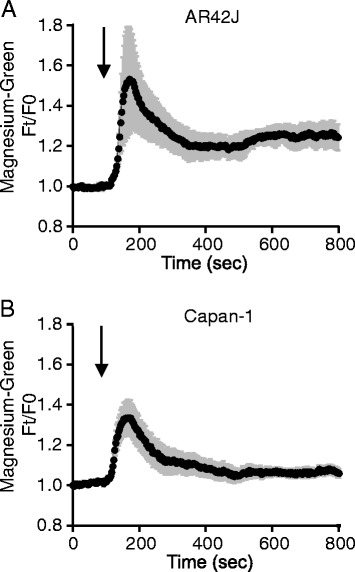
CDCA effect on Magnesium Green fluorescence in AR42J and Capan-1. Effect of 0.5 and 0.3 mM CDCA on intracellular ATP (ATP_i_) changes in AR42J (**a**) (n = 3) and Capan-1 (**b**) (n = 4) cells. Cells were loaded with Magnesium Green indicator (5 μM) for 20 min. Changes of ATP_i_ are given as ratios of fluorescence at time t in relation to time 0 (F_t_/F_0_), where the starting baseline is set to the 1. Results are shown as mean values ± SEM of 10 cells or group of acini per each individual experiment. Arrows indicate time of adding CDCA

Since we observed that CDCA stimulated substantial ATP release from exocrine pancreatic cells, in the following experiments we investigated whether this was accompanied by a decrease of intracellular ATP concentrations, ATP_i_. We used the Magnesium Green (MgGreen), an indirect ATP sensor, as it increases in fluorescence when ATP concentrations decrease and the released Mg^2+^ can bind to the fluorophore. After CDCA stimulation, there was a fast and transient increase in the MgGreen F/F0 fluorescence ratio to 1.53 ± 0.21 in AR42J (n = 3), and then the ratio decreased but remained elevated by about 0.2 units above the basal value (Fig. 3a). Capan-1 cells also responded by a transient increase in F/F0 ratio to 1.34 ± 0.08 (n = 6), but after 300–400 s the signal recovered to pre-CDCA values (Fig. 3b). The transient changes in MgGreen fluorescence could have several explanations (see Discussion). Since we observed a tendency to partial depletion of ATP_i_ in AR42J cells after prolonged stimulation with CDCA (Fig. 3a), we used a more direct method employing one of the ATeam sensors developed by Imamura and colleagues [41].

**Fig. 4:**
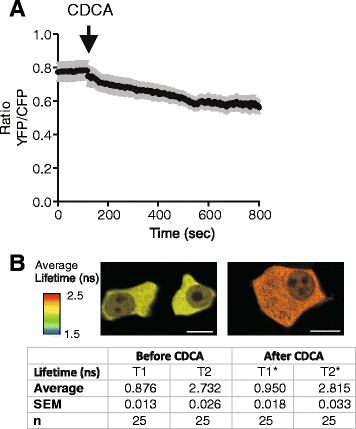
Effect of CDCA on ATP sensor (AT1.03^YEMK^) in AR42J cells. **a** Effect of 0.5 mM CDCA on FRET ratio of the ATP sensor in AR42J cells (n = 25). **b** Lifetime images of cells before and after treatment with CDCA. Images, where each pixel was analysed, are average of the two lifetime constants corrected for intensity scale. The insert table summarizes FLIM-FRET life constants T1 and T2 in 25 independent experiments. * *P* = 0.0001 for T1 and *P* = 0.007 for T1 and T2 comparison

AR42J cells were transfected with AT1.03^YEMK^ and simultaneous images of YFP and CFP were used to build YFP/CFP ratio. Fig. 4a shows that there was a small decrease in the ratio with CDCA incubation, which indicates decreased FRET. In addition, in separate fluorescence lifetime imaging (FLIM) experiments we determined CFP fluorescence lifetime of AT1.03^YEMK^ using the time-correlated single-photon counting technique. The data were used to generate a lifetime map by fitting each point to a double exponential decay. *χ*^2^ values were lying around 0.9-1.1. The sensor had a uniform lifetime distribution within the cell cytoplasm (Fig. 4b). It is well established that CFP has bi-exponential decay and the reported lifetimes lie around 1.1–1.3 ns and 2.8–2.9 ns [42]. For untreated AR42J cells we found two lifetimes of 0.876 ± 0.013 ns and 2.732 ± 0.026 ns (n = 25). These lifetimes were shorter than the reported lifetime for CFP due to fact that the interacting donor lifetime is shortened by ATP binding and causing FRET in the AT1.03^YEMK^ sensor. After addition of CDCA the lifetimes increased to 0.950 ± 0.018 and 2.815 ± 0.033 in the same experiments, indicating that there is less FRET, likely corresponding to less ATP within the cell.

**Fig. 5:**
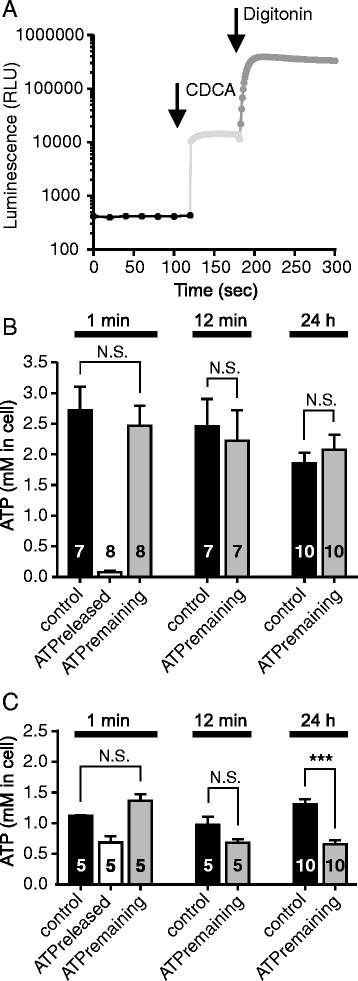
The acute and chronic effects of CDCA on intracellular ATP concentration in AR42J and Capan-1 cells. **a** Original trace for luminometric measurements of ATP based on Capan-1 measurement. Baseline values were recorded every 20 s for 2 min. Cells were then stimulated with 0.3 mM (Capan-1) or 0.5 mM (AR42J) with CDCA or with vehicle. Stimulated ATP release was recorded every 1 sec for 1 min directly after addition of CDCA, or after 12 min or 24 h of incubation. Finally, cells were permeabilized with digitonin (50 μM), added automatically using pump to release remaining ATP. Panel **b** and **c** show the values of released and remaining ATP in Capan-1 and AR42J cells, respectively. These values were calculated per cell after 1, 12 min and 24 h (n = 8, 7, 10 and n = 5, 5, 10) of incubation with CDCA. Data are shown as mean values ± SEM; ***= *P* < 0.001, N.S. - not significant

The above ATP_i_ measurement methods are dynamic, but difficult to calibrate in acinar cells. Therefore, we also used luciferin/luciferase assay to determine ATP_i_. Cell membranes were permeabilized with digitonin after CDCA treatment and intracellular ATP_i_ was quantified (Fig. 5a). ATP_i_ concentrations were measured at different time points (1 and 12 min), correlating with the peak and plateau for MgGreen (Fig. 3). Additionally, the long-term effect was also determined by incubating the cells with CDCA for 24 h. After stimulation with 0.3 mM CDCA, Capan-1 released 68 ± 26 nM ATP to extracellular medium, which corresponds to a calculated decrease of 0.08 ± 0.03 mM ATP_i_ in a cell (n = 8). Fig. 5b shows that after incubation of duct cells for 1 min and 12 min, the total content of remaining ATP_i_ was not significantly changed and remained at 2.47 ± 0.32 mM calculated per cell; (n = 8) and 2.22 ± 0.5 mM (n = 7) compared to their respect controls of 2.72 ± 0.38 mM and 2.46 ± 0.45 mM (n = 7). Furthermore, the long-term exposure of Capan-1 to 0.3 mM CDCA did not cause significant changes in ATP_i_ concentrations, i.e., 2.08 ± 0.24 mM compared to control 1.85 ± 0.18 mM (n = 10). For AR42J cells, stimulation with 0.5 mM CDCA (Fig. 5c) caused ATP release of 358 ± 52 nM to extracellular medium, which corresponds to a calculated decrease of 0.68 ± 0.1 mM in the cell (n = 5). Similar to the Capan-1 cells, we did not observe significant changes in remaining ATP_i_ concentrations after 1 min (1.36 ± 0.1 mM; n = 5). However, after 12 min there was a tendency, though not significant, of lower intracellular ATP levels (0.68 ± 0.06 mM; n = 5) with CDCA compared to their respective controls (1.12 ± 0.01 mM and 0.97 ± 0.13 mM; n = 5). Furthermore, after 24 h incubation of AR42J with 0.5 mM CDCA, there was a significant decrease of ATP_i_ to 0.66 ± 0.06 mM compared to the control 1.3 ± 0.08 mM (n = 10).

### CDCA-induced intracellular Ca^2+^ responses are inhibited by P2 receptor inhibitors

**Fig. 6:**
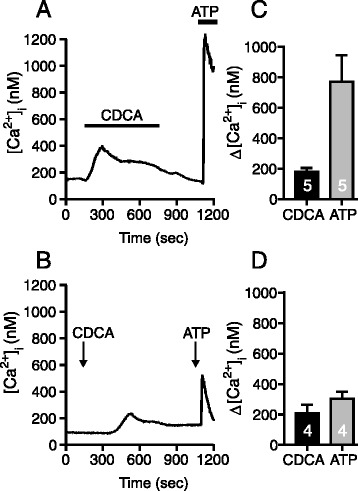
Effect of CDCA and ATP on [Ca^2+^]_i_ responses in perfused and non-perfused conditions in Capan-1 cells. Representative recordings of intracellular Ca^2+^ concentrations [Ca^2+^]_i_ in Capan-1 cells in perfused and non - perfused (standing bath) conditions. **a** A chamber with Capan-1 cells was perfused (1 ml/min) with physiological buffer containing 0.3 mM CDCA or 100 μM ATP. **b** Addition of CDCA to a standing bath showed delayed response to CDCA and diminished [Ca^2+^]_i_ response induced by ATP (100 μM). **c** and **d** show the summary of data as mean values ± SEM, (n = 5, 4)

Bile acids are reported to increase [Ca^2+^]_i_ in pancreatic cells, but it is not clear which receptors are involved (see Introduction). Since we observed that CDCA induced ATP release, we hypothesized that the intracellular Ca^2+^ responses could be due to effect of released ATP on P2 receptors, which are well established regulators of stimulated Ca^2+^ influx and/or Ca^2+^ release in pancreatic ducts. Therefore, [Ca^2+^]_i_ was monitored in Capan-1 cells in a chamber perfused with physiological solution containing CDCA followed by ATP (Fig. 6a,c). CDCA evoked a slow Δ[Ca^2+^]_i_ increase (180 ± 24 nM, n = 5). Similar slow and small responses to other BAs were also reported for pancreatic acinar cells and cholangiocytes [25, 43]. In contrast, infusion of ATP caused fast and markedly higher Δ[Ca^2+^]_i_ increase (772 ± 173 nM, n = 5). Further experiments were conducted without perfusion, in order to minimize mechanical stimulation and amount of inhibitors used. In standing bath CDCA evoked similar Δ[Ca^2+^]_i_ response as in the perfused conditions, but the signal was delayed (Fig. 6b, d). Stimulation with ATP in the presence of CDCA, caused a lower but fast Δ[Ca^2+^]_i_ response (302 ± 47 nM, n = 4), perhaps because P2 receptors were already desensitized by CDCA-induced ATP release as ATP was not washed away during perfusion. Therefore, for the following studies with P2R inhibitors, we conducted separate experiments for the ATP and CDCA stimuli.

**Fig. 7:**
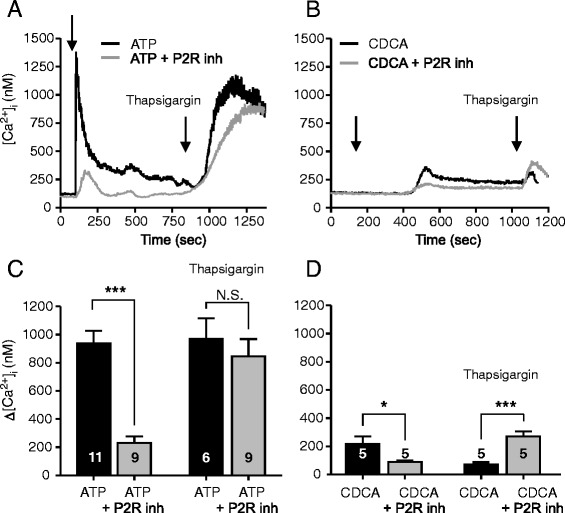
Effect of P2 receptor inhibitors on intracellular Ca^2+^ responses induced by CDCA in Capan-1 cells. **a** and **b** Representative recordings of [Ca^2+^]_i_ transients in Capan-1 cells with or without P2 receptors inhibitors (standing bath). Cell were incubated with a mix of P2R inhibitors: PPADS (250 μM), suramin (250 μM), and 10 μM of AZ 10606120 and 10 μM of A438079 for 25 min. **a**, **c** The presence of P2R antagonists markedly inhibited [Ca^2+^]_i_ response induced by ATP (100 μM), but had no effect on Thapsigargin (1 μM) induced Ca^2+^ response. **b**, **d** Incubation cells with P2R antagonists inhibited [Ca^2+^]_i_ transient induced by 0.3 mM CDCA but not by Thapsigargin (1 μM). **c**, **d** Change in [Ca^2+^]_i_ above baseline are given as mean values ± SEM of 7–15 cells per each independent experiment (n). Arrows indicate the time of adding the stimuli. *= *P* < 0.05, ***= *P* < 0.001, N.S = not significant

Figure 7a-d shows that the P2R inhibitors markedly reduced the ATP-stimulated Δ[Ca^2+^]_i_ response from 937 ± 88 nM (n = 11) to 232 ± 47 nM (n = 9). The antagonists also caused significant inhibition of CDCA stimulated Δ[Ca^2+^]_i_ (from 217 ± 54 to 90 ± 9 nM; n = 5). These data indicate that the Δ[Ca^2+^]_i_ response evoked by the bile acid could be a result of P2 receptor stimulation by CDCA-induced ATP release. Thapsigargin, the inhibitor of SERCA [44], was added at the end of the experiments to inhibit re-uptake of Ca^2+^ to intracellular stores. Thapsigargin induced a small Δ[Ca^2+^]_i_ increase after CDCA by 70 ± 17 nM (n = 5) but a large increase after ATP, i.e., 971 ± 143 nM, (n = 6). P2R inhibitors did not have effect on intracellular Ca^2+^ response induced by thapsigargin after ATP (Fig. 7c). Interestingly, when thapsigargin was applied after CDCA in the presence of the P2R inhibitors (Fig. 7d), Δ[Ca^2+^]_i_ was significantly higher (270 ± 35 nM, n = 5).

### Expression of the TGR5 and FXR receptors in exocrine pancreatic cells

**Fig. 8:**
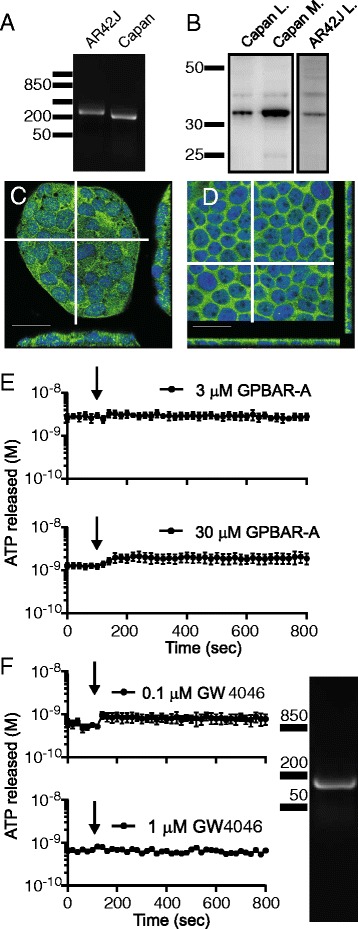
TGR5 and FXR receptors expression in exocrine pancreatic cells. **a** RT-PCR and **b** Western Blot analysis of the TGR5 receptor expression in AR42J and Capan-1 cells, in lysates (L) and membrane microdomain enriched fraction (M) shows a clear band at 33 kDa. **c** - **d** Immunocytochemistry of TGR5 in AR2J and Capan-1 cells. Scale bars are 25 μm. White lines indicate where the z scan was taken. **e**. The effect of the TGR5 receptor agonist GPBAR-A at 3 and 30 μM (n = 3, 4) on ATP release from Capan-1 cells. **f** Expression of FXR in Capan-1 cells showed by RT-PCR. Activation of FXR with the specific agonist GW4046 at 0.1 and 1 μM (n = 3, 2) did not have effect on ATP release from duct cells. Arrows indicate when the agonists were added

It is not known whether TGR5 and FXR receptors are expressed in the human pancreatic ducts and could account for the effects seen in our study. Also from studies on animal pancreatic tissue, it is not certain whether TGR5 is expressed in ducts [25, 29]. We therefore investigated TGR5 expression in AR42J and Capan-1 cells using RT-PCR and Western Blot. Fig. 8a, b shows that TGR5 is expressed in both cell lines. This observation was confirmed by immunostaining of the acinar cells and on a polarized monolayer of Capan-1 (Fig. 8c, d). In duct epithelium it appears that the receptor is localized mainly on the luminal membrane. Since TGR5 is expressed in pancreatic cells, we wanted to determine whether the receptor has any effect on ATP release and for this purpose we used GPBAR-A, a specific TGR5 receptor agonist that upon binding stimulates cAMP synthesis [45]. Using the luminescence method, we did not detect ATP release (Fig. 8e) from Capan-1 cells after stimulation with 3 or 30 μM GPBAR-A.

In another series of experiments we investigated whether Capan-1 cells express the nuclear type of bile acid receptor, FXR, and whether its stimulation might induce ATP release. We indeed found the transcript for FXR in Capan-1 cells using RT-PCR (Fig. 8f). However, activation of the receptor with the specific agonist GW4064 (0.1 and 1 μM) did not cause significant ATP release (Fig. 8f). Based on these observations, we concluded that stimulation of BAs receptors TGR5 and FXR with specific pharmacological agonists does not induce ATP release from duct cells.

**Fig. 9:**
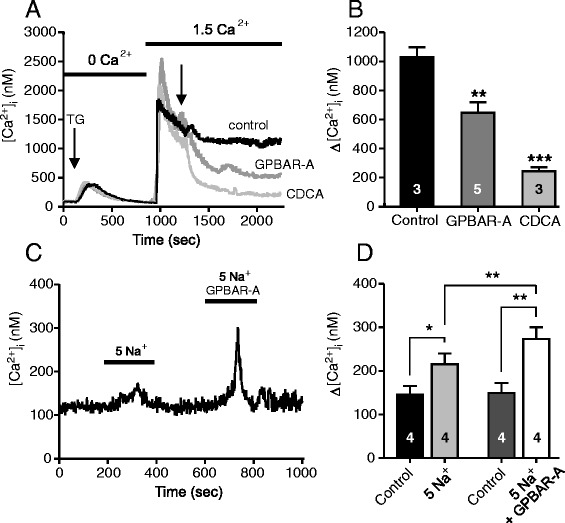
Effect of the TGR5 ligands on [Ca^2+^]_i_ transients in duct epithelia. Capan-1 cells were incubated with nominal 0 mM Ca^2+^ buffer (**a**, **b**) and thapsigargin (1 μM) to deplete intracellular Ca^2+^ stores. Thereafter, cells were gently perfused with physiological buffer to refill intracellular Ca^2+^ stores and after the fluorescence was relatively stable, solutions were changed to GPBAR-A (30 μM), CDCA (0.3 mM), or control. **c**, **d** The contribution of the sodium-calcium exchanger (NCX) was tested. Cells were perfused with 5 mM Na^+^ buffer which increased [Ca^2+^]_i_ and this response was potentiated in the presence of GPBAR-A (30 μM). **b**, **d** Summary of data given as mean values ± SEM of 7–15 cells per each independent experiment (n). *= *P* < 0.05, ***= *P* < 0.001, N.S = not significant

Above we observed that CDCA appeared to protect Capan-1 cells from thapsigargin effects (Fig. 7d, f). This could occur if, for example, CDCA stimulated SERCA (or protected the pumps from thapsigargin) or if CDCA stimulated alternative Ca^2+^ efflux pathways. Our hypothesis was that the TGR5 receptor could be involved in these effects and we tested this in the following experiments. First, intracellular Ca^2+^ stores were emptied by thapsigargin in low Ca^2+^ medium (Fig. 9a). Thereafter, extracellular Ca^2+^ was reintroduced, and since SERCA pumps were inhibited, [Ca^2+^]_i_ increased to very high levels in control cells. Notably, perfusion of the cells with CDCA caused a fast and marked reduction in [Ca^2+^]_i_ from 1253 ± 117 to 244 ± 28 nM (Fig. 9 a, b, n = 3). A similar response was observed with perfusion of GPBAR-A (Fig. 9a-b), which lowered the [Ca^2+^]_i_ from 1249 ± 69 nM to 647 ± 71 nM (n = 5).

In next experiments we addressed the question whether the lowering of [Ca^2+^]_i_ caused by CDCA could involve Ca^2+^ efflux via the Na^+^/Ca^2+^ exchanger (NCX), which is expressed in duct epithelia [46, 47]. Since CDCA evokes Ca^2+^ transients itself, we used, GPBAR-A, which showed no effect on Ca^2+^ responses when given alone (see Additional file 1: Figure S1). Capan-1 cells were perfused with a buffer containing 5 mM Na^+^, which favours Ca^2+^ influx via NCX as observed in Fig. 9c. This effect of low Na^+^ on [Ca^2+^]_i_ was increased with GPBAR-A (30 μM), which is consistent with TGR5 stimulation of NCX.

## Discussion

The present study shows that some of the BA (i.e., CDCA) effects are due to stimulation of purinergic signalling. Firstly, acini and ducts (AR42J and Capan-1 cells) release substantial amounts of ATP in response to CDCA, and this process can be inhibited by several inhibitors of ATP release. Secondly, CDCA effects on intracellular Ca^2+^ could be significantly inhibited by P2 receptor antagonists, indicating cross-activation of the two signalling pathways. Furthermore, the TGR5 receptor affected intracellular Ca^2+^ indirectly by activating NCX. Below, we discuss these results and propose that interplay between BAs and purinergic signalling may be important in physiological regulation of pancreatic function.

In order to evaluate physiological or pathological effects of BAs, it is important to consider what BAs concentrations pancreas may encounter. In healthy human, plasma BAs concentrations are <5 μM in resting state and about 10–15 μM post-prandial. However, in case of liver or pancreatic diseases, BAs concentrations can increase to around 300 μM [48, 49]. In normal bile, the concentration of bile acids is higher than 100 mM, and CDCA contributes to about 50 % [50]. Therefore, pancreas could be exposed to low BA concentrations as well as to higher concentrations resulting from bile reflux into pancreatic duct tree following outflow obstruction, e.g., gallstones. In the current studies, we used concentrations that have been considered stimulatory on pancreatic ducts [27, 28].

The most important finding is that primary bile acids can cause ATP release and therefore stimulate purinergic signalling in exocrine pancreatic cells. The unconjugated CDCA had the most pronounced effects compared to far less effective glycine- and taurine-conjugated forms of the acid (Fig. 1). Thus CDCA caused fast and large ATP release from both acinar and duct cells (Fig. 1). In comparison, cholinergic or hormonal stimulation or cell swelling of pancreatic cells induced significantly lower ATP release [34, 35]. We investigated the mechanisms of ATP release and find that both exocytotic pathways and ion channels/receptors seemed to be involved in this process (Fig. 2b, c). Our findings with bafilomycin, brefeldin and NEM strongly support participation of a vesicular component in ATP release, e.g., VNUT (Fig. 2b), which is also expressed in duct cells (unpublished data). In addition, the inhibitor data (Fig. 2C) indicate that connexin hemichannels and/or pannexin with P2X7R could also be involved in ATP release. One of the most potent blockers was Gd^3+^ that can inhibit pannexin/connexins, Ca^2+^ influx and hence exocytosis, and it is known inhibitor of maxi-anion channels which may be a part of osmosensitive ATP release with hypotonic stress [51, 52]. We cannot exclude that CDCA either can activate these maxi-anion channels directly or it induce cell volume changes, though cell volume changes due to hypotonic shock (unpublished data) have much smaller impact on ATP release than CDCA. The P2X7 receptors may also have positive effects on CDCA-induced ATP release (Fig. 2c), and modulating effects of the receptor on exocytosis and/or pannexin-1 have been described in other cells [53, 54]. CFTR has also been proposed as a channel/regulator for ATP release [36] and in bile ducts the secondary ursodeoxycholic acid stimulates CFTR-dependent ATP secretion [43]. In Capan-1 cells, CFTR does not seem to contribute to ATP release (unpublished data). Taken together, we propose that several ATP releasing mechanism contribute to CDCA-evoked ATP release, and result in extracellular ATP increase by 100–1000 fold, which is the largest increase compared to that observed with other stimuli (unpublished data).

One important point to consider was how the BA-induced ATP release is triggered. Since GPBAR-A and GW4046 had no effects on ATP release, we presume that the TGR5 and FXR receptors are not involved. An alternative mechanism could be BA induced membrane depolarisation [21], which may be mediated by activation of bile acid-sensitive ion channel (BASIC), which belongs to the DEG/ENaC family, recently identified in bile ducts [55]. In addition, CDCA may incorporate into membranes, increase membrane fluidity [56] and thereby affect one or more of ATP release mechanisms as proposed above. Nevertheless, we show that the ATP release is clearly directed towards the lumen of pancreatic ducts (Fig. 2a), and therefore it is likely that there is a trigger mechanism and luminal exocytosis/transport mechanisms. Also in other epithelia, e.g., kidney tubuli and airway epithelia, ATP is released preferentially to the apical/luminal side in response to a number of stimuli [57–60].

Regarding effect of BA in pancreas, we propose that BA stimulates release of ATP towards lumen and then ATP binds to P2 receptors and therefore stimulates Ca^2+^ signalling pathways, and these can potentially increase Cl^−^ and K^+^ conductances, which are necessary to initiate and acid/base transport and thus ductal fluid secretion. Indeed it is well documented that several P2 receptors regulate ion channels such as TMEM16A/ANO1, CFTR and K_Ca_3.1 and K_Ca_1.1 [32, 61]. The more traditional view is that BAs acting directly on BA receptors can affect epithelial transport [10, 26, 62]. For example, in airway epithelial cells, taurodeoxycholic acid (TDCA) stimulates CFTR and Ca^2+^-activated Cl^−^ currents and these effects seems to be mediated by the basolateral TGR5 receptor [10]. From published studies on pancreatic duct epithelia, it was not clear whether BA receptors were expressed. Nevertheless, it is reported that in dog pancreatic duct epithelial cells TDCA also increased Cl^−^ and K^+^ fluxes [26], and in guinea pig pancreatic ducts BAs stimulate K_Ca_1.1 channels, but accompanying Cl^−^ channels have not been detected [27, 30].

Previous studies show that CDCA (0.5 mM) caused ATP depletion in pancreatic acinar cells and colon epithelial cells, presumed to be due to inhibited metabolism [63, 64]. Since CDCA induced such a large ATP release in our exocrine cells (Fig. 1), it was relevant to examine whether CDCA could also affect intracellular ATP. We have used several techniques to study this, including intracellular ATP sensors. The transient increase in MgGreen fluorescence could indicate transient decrease in ATP_i_. However, since CDCA and ATP caused transient increase in [Ca^2+^]_i_, and the fluorophore can also bind Ca^2+^, the MgGreen signals can have several components. In addition, in AR42J cells there was a slow increase in MgGreen fluorescence occurring long after Ca^2+^ peak. There was a similar slow effect of CDCA on AT1.03^YEMK^ ratio, as well as a change in the lifetime constants of the sensor. These data together indicate that there was a decrease in ATP_i_. Using luciferase assay, we find that in the first minute after CDCA stimulation acinar cells release significant amount of cellular ATP (Fig. 5c), though ATP_i_ seems to decrease first after 12 min, as also indicated by the AT1.03^YEMK^ measurements (Fig. 4). Nevertheless, it is only after long-term incubation of AR42J cells with 0.5 mM CDCA that significant depletion in ATP_i_ was detected by luminescence assay (Fig. 5c). This depletion could be due to decreased mitochondrial ATP production and/or secondary effects caused by digestive enzymes released by CDCA-induced exocytosis [20, 63, 65] and by exocytosis/release of ATP, as we show in the present study. In contrast to acini, pancreatic ducts seem very robust. In duct cells, CDCA caused release of less than 3 % of total intracellular ATP, and ATP was presumably replenished (Fig. 5b), which also agrees with total recovery of MgGreen fluorescence with continued CDCA stimulation. Interestingly, in pancreatic islets, BAs (e.g., tauroursodeoxycholate) are not detrimental but enhance ATP_i_ concentration [15].

It is extensively documented that BAs (0.1 – 1 mM) cause an increase in [Ca^2+^]_i_ in many cells, including pancreatic cells and mechanisms include increased release from ER, inhibition of SERCA and increased Ca^2+^ entry [20, 21, 27, 66]. Regarding TGR5, it is well established that the receptor interacts with G_s_ protein and leads to stimulation of adenylate cyclase and cAMP signalling [5, 12, 45, 67]. In addition, several reports show that BAs (high μM concentrations) show small and slow increases in intracellular Ca^2+^ [10, 18, 21, 23, 25, 45]. Underlying mechanisms are unclear, as seen in a number of studies, though one study has suggested the involvement of TGR5 [10, 18, 21, 23, 25, 45]. In pancreatic acinar cells genetic deletion of TRG5 still leaves BA-induced Ca^2+^ transients in many cells and BA-stimulated amylase release is relatively undisturbed [25], indicating that there is not very tight coupling between TGR5 and Ca^2+^ signalling. Since GPBAR-A had no effect on [Ca^2+^]_i_ in our duct cells expressing the receptor, it seems that TGR5 receptor is not involved in initiating simple Ca^2+^ transients in given experimental conditions. We propose that CDCA-evoked ATP release leads to P2 receptor activation and thereby increases in [Ca^2+^]_i_. Indeed, CDCA-stimulated Ca^2+^ increase was inhibited by a cocktail of P2R antagonists (Fig. 7d). This observation indicates that a significant part of Ca^2+^-dependent BA effects could be due to ATP release and subsequent stimulation of P2 receptors expressed in duct cells. These processes and potential effects on ion transport are acute (seconds to minutes). Our study did not address the question whether BAs via activation of FXR and TGR5 could regulate purinergic receptor expression on a longer time-scale.

The differences in the thapsigargin induced Ca^2+^ response after stimulation with ATP or CDCA lead us to speculate whether there is a possible protective role of CDCA during high [Ca^2+^]_i_ stress conditions in pancreas. It has been shown that CDCA can protect cells when ER is depleted of Ca^2+^ [68]. We observed that CDCA markedly decreased the thapsigargin-induced high [Ca^2+^]_i_ and that the GPBAR-A was similarly effective (Fig. 9a). Others have shown that bile acids could prevent thapsigargin-evoked ER stress in liver, adipocytes and β-cells [15, 69]. Our observations are in the line with these and we suggest that the “protective effect” of CDCA is mediated via TGR5 activation. Pancreatic ducts express NCX, which is stimulated by cAMP and Ca^2+^ [47]. We propose that some of the CDCA-induced decrease in high [Ca^2+^]_i_ conditions could be due to NCX activation. Indeed stimulation of TGR5 with GPBAR-A increased NCX activity (Fig. 9c). It should be noted that since NCX is electrogenic (exchanging 1 Ca^2+^ : 3 Na^+^), it can transport Ca^2+^ in or out of the cell, depending in electrochemical potential, and this could vary depending on the cell type and its stimulation.

## Conclusions

**Fig. 10:**
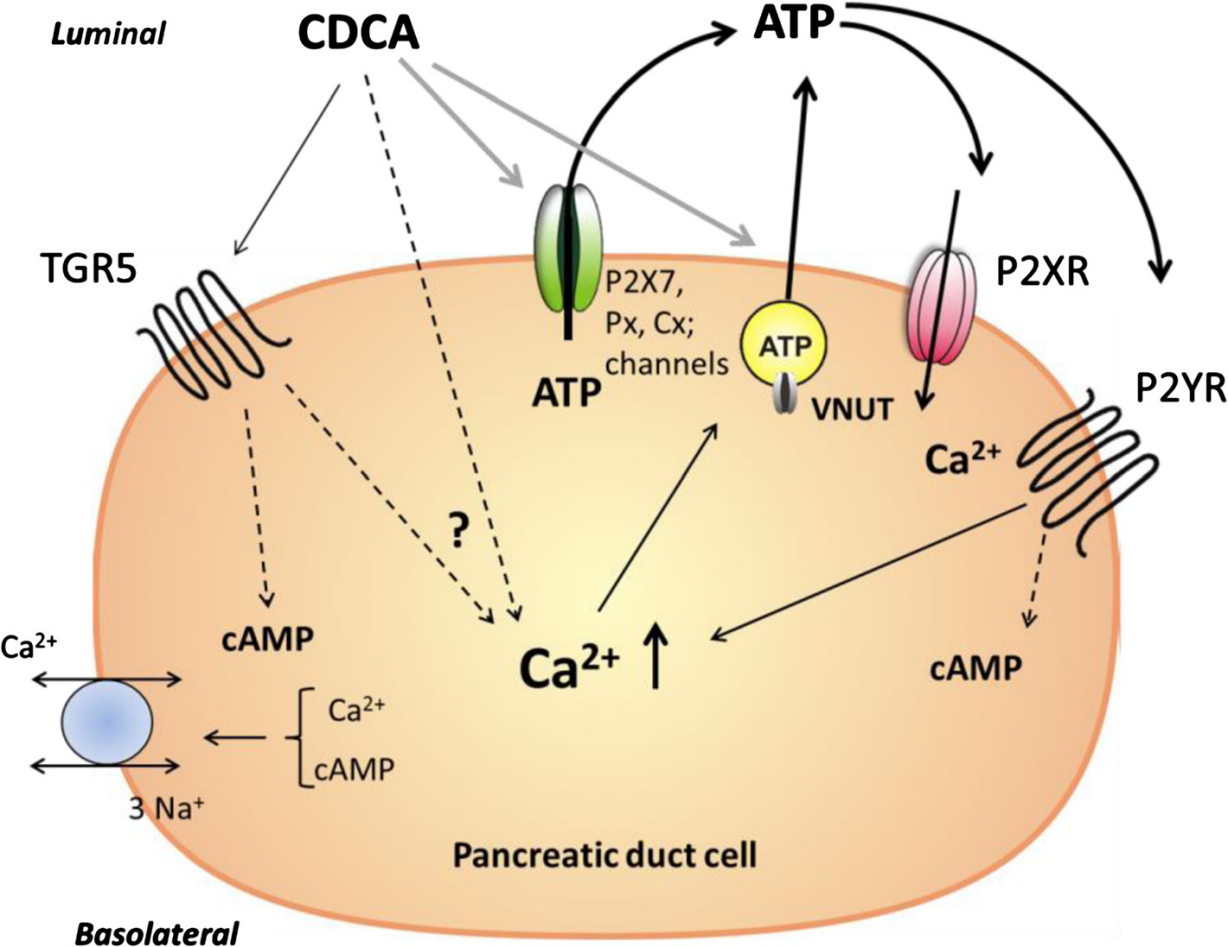
Proposed model of CDCA-induced cellular responses in pancreatic duct cells. CDCA acting on the plasma cell membrane induces ATP release via non-vesicular releasing mechanisms (pannexin, connexin, P2X7R, other ion channels) and vesicular exocytosis of ATP. Released ATP may in turn activate P2X receptors/cation channels and P2Y receptors, which allow Ca^2+^ influx directly (P2XR) or via G-protein coupled signalling mediate Ca^2+^ release from intracellular stores and Ca^2+^ influx (P2YR). Furthermore, CDCA may activate TGR5 receptor, which leads to stimulation of adenylyl cyclase and cAMP production, possibly increase in Ca^2+^ in some cells by yet undefined mechanisms. The sodium-calcium exchanger (NCX) can be stimulated by TGR5 resulting of transport of Ca^2+^ out of the cell, or into the cell, depending on prevailing electrochemical gradients

In conclusion, as summarized in Fig. 10, the most important finding in our study is that unconjugated BA evokes significant ATP release from pancreatic exocrine cells, this ATP in turn can stimulate P2R, which thereby increases [Ca^2+^]_i_. We show the expression of the TGR5 receptor in a human duct cell line, where it could play a protective role at high intracellular Ca^2+^ conditions. Taken together, for pancreas we envisage that purinergic signalling is a significant part of the cellular response to BA and could support the physiological function such as secretion. Finally, we propose that purinergic signalling, i.e., ATP release and cell/organ P2 receptor involvement, should be taken into consideration in other cell/organ types, as it could potentially explain the multifaceted and ubiquitous effects of BAs.

## Methods

### Chemicals

All chemicals were purchased from Sigma-Aldrich unless otherwise specified. In present study following chemicals were used: chenodeoxycholic acid (CDCA sodium salt, 0.1 – 1 mM), glycochenodeoxycholic acid (GCDCA sodium salt, 0.3 and 1 mM), taurochenodeoxycholic acids (TCDCA sodium salt, 0.3 and 1 mM), ATP (100 μM), digitonin (50 μM), thapsigargin (1 μM), 4-[[3,5-Bis(trifluoromethyl)phenyl]methyl]-6-(2-fluorophenyl)-4,5-dihydro-pyrido[3,2-*f*]-1,4-oxazepin-3(2*H*)-one (GPBAR-A, 30 μM, Tocris) dexamethasone (50 nM). Cells were pre-treated/incubated with inhibitors and fluorescent indicators as follow: bafilomycin A1 (1 μM), N-Ethylmaleimide (NEM, 250 μM), brefeldin A (5 μg/ml; Molecular Probes- Life Technology), pannexin inhibitor ^10^Panx (100 μM, Tocris), gadolinium chloride (Gd^3+^, 50 μM), probenecid (500 μM), P2X7 inhibitors: AZ10606120 (10 μM, Tocris) and A438079 (10 μM, Tocris), pyridoxal phosphate-6-azo(benzene-2,4-disulfonic acid) tetrasodium salt hydrate (PPADS, 250 μM), suramin 250 μM, Magnesium Green indicator (MgGreen; 5 μM, Invitrogen), Fura-2 AM calcium indicator (5 μM, Teflabs), pluronic F127 (Molecular Probes- Life Technology), ATP kit SL 144–041 (BioThema), cell counting kit-8 (CCK-8, DOJINDO).

### Cell cultures

Pancreatic rat acinar (AR42J, CRL-1492) and human duct (Capan-1, HTB-79) cell lines were obtained from ATTC (Manassas, VA) and cultured according to recommended procedures. For imaging experiments WillCo-dishes (WillCo Wells BV, Amsterdam, the Netherlands) were used and for luminescence, 96-well NUNC white plates. For luminescence recordings of ATP release, 50,000 Capan-1 cells were cultured to 80 % confluence and for imaging 35.000 cells were cultured for 2 days. For all experiments for AR42J, 10,000 cells (or 40,000 cells for FRET-FLIM analysis) were plated and grown for 48 h, followed by 48 h with 50 nM dexamethasone to induce an acinar phenotype, which increases formation of zymogen granules [70]. All experiments were conducted at 37 °C.

### Measurement of ATP concentrations

Different protocols were used to measure ATP: in extracellular fluid; in single cells; permeabilized cells and polarized cells. Extracellular ATP (ATP_e_) was monitored in using luciferase + luciferin luminescence reaction in extracellular medium. Capan-1 and AR42J cells were washed and allowed to rest in 65 μl of physiological buffer that contained (in mM): 140 NaCl, 1 MgCl_2_ · 6H_2_O, 1.5 CaCl_2_, 0.4 KH_2_PO_4_, 1.6 K_2_HPO_4_ · 3H_2_O, 10 HEPES and 20 glucose (Capan-1) or 10 glucose (AR42J); pH = 7.4. After 30 min, 25 μl of 2 × concentrated luciferase/luciferin mix was added into the wells. Measurements were made using plate mode (20-s sampling rate with 1-s integration) in FLUOstar Optima (BMG, Labtech). CDCA (10 μl) was gently added manually. To quantify CDCA-induced intracellular ATP (ATP_i_), similar protocol was repeated but after 1 min of recordings, cells were permeabilized with 25 μl of digitonin (50 μM). For each experimental protocol, standard curves were made with ATP concentrations 10^−9^ to 10^−5^ M. The bile acids were included in all standards. Also the effect of the inhibitors on luciferase/luciferine was tested independently on standard curves (Additional file 1: Figure S2 a-b). Cell number was determined by cell counting kit and ATP concentrations were corrected for 10^6^ cells per 1 ml. To calculate the single cell ATP_i_ concentration, volumes of Capan-1 and AR42J were estimated assuming a spherical shape with diameter of 12 μm and 10 μm, respectively.

To investigate the polarity of ATP release, i.e., to the luminal or basolateral sides of polarized epithelium, 200,000–400,000 Capan-1 cells/cm^2^ were seeded on collagen-coated Snapwells (no. 3407; Corning). When resistance of the monolayer was ~ 500–600 Ω_*_cm^2^, Snapwells were put into a holder, and monolayers were incubated with 500 μl of physiological solution on each side (luminal and basolateral). 150 μl of fluid was collected for a baseline value, and cells were then luminally or basolaterally stimulated with 0.3 mM CDCA. 150 μl samples collected from apical and basolateral sides and heated for 1 min in 98 °C and kept on ice before analysed for ATP.

### Magnesium-Green fluorescence measurement

ATP_i_ was estimated indirectly using Magnesium Green (MgGreen) fluorescence. Cells were incubated with 5 μM MgGreen in presence of 0.02 % pluronic F127 for 20 min and gently washed 15 min before experiments. MgGreen was excited with an argon laser (488 nm) and emission was monitored at 498–581 nm using a confocal laser scanning microscope (TCS SP 5X, Leica Microsystems, Heidelberg). Images were captured every 3 sec using a 20X N.A. 0.7 immersion (water) objective. Images analysis was performed on 10 single cells or group of AR42J per dish. Changes of ATP_i_ responses are presented as ratios at time t in relation to time 0 (F_t_/F_0_) and baseline is set to 1.

### ATP sensor FRET-FLIM microscopy

ATP_i_ was estimated more specifically using FRET sensor based on ε subunit of bacterial F0F1-synthase, i.e., AT1.03^YEMK^ [41]. AR42J cells were transfected using FuGENE transfection kit (Promega) according to manufacturer’s protocol. Media was changed the day after transfection to media containing dexametasome. Experiments were conducted on AR42J cells bathed in physiological saline containing 10 mM glucose and the temperature was 37 °C and images were collected with a 63 x water objective (HCX PLAPO, 1.2 NA) in a TCS SP5X Confocal microscope). Two types of recordings were made: FRET ratio analysis and FRET-FLIM analysis. In time resolved protocols, 405 nm laser was used for excitation of the CFP, the fluorescence of CFP and excited YFP was collected simultaneously at 460–500 nm for CFP and 530–570 nm for YFP. Images were taken at 3 s intervals. The individual measurements were corrected for background and YFP/CFP ratio was continuously recorded. In separate experiments, FRET was estimated using fluorescence lifetime imaging (FLIM). The SP5X microscope, equipped with a time-correlated single-photon counting (TCSPC) module (PicoHarp 300, PicoQuant, Berlin, Germany), allows FLIM analysis using a Mai-Tai Ti-Sapphire laser (850 nm) providing 80 ps pulses. The CFP lifetime was recorded with the emission filter: 450–500 nm and detected by FLIM PMT. The laser power was reduced so the counting was kept under 800 kcounts to avoid build up effects. Photons were collected continuously for 2 min for each image. The decay curve was analysed with the SymProTime software (PicoQuant) via a tail-fitting procedure. The counts around the peak were typically around 10^3^ photons and the quality of the fit was judged on the basis of the chi-squared statistic, *χ*^2^, and reduced randomness of residuals. Three to five different positions in each dish with cells were analysed before and after addition of CDCA.

### Calcium signals

Capan-1 cells were incubated with Fura-2 AM in the presence of 0.02 % pluronic F127 and probenecid (0.25 mM) in physiological buffer for 30 min. For perfusion experiments the perfusion rate was 1 ml/min. For standing bath (non-perfused) conditions cells were incubated for 25 min with or without P2R antagonists. In some experiments, Capan-1 cells were stimulated with Thapsigargin in nominal Ca^2+^-free buffer containing (in mM): 115 NaCl, 25 Na-gluconate, 1 MgCl_2_ · 6H_2_O, 5 HEPES, 1.6 K_2_HPO_4_ · 3H_2_O, 0.4 KH_2_PO_4_, 20 glucose, 5 EGTA. Subsequently, cells were gently perfused with buffer containing Ca^2+^ and after 6–8 min cells were perfused with physiological buffer including CDCA or GPBAR-A. In order to examine the role of the sodium-calcium exchanger (NCX) on [Ca^2+^]_i_ changes, Capan-1 cells were perfused with 5 mM Na^+^ buffer, where the rest of NaCl was substituted with 140 N-Methyl-D-Glucamine (NMDG) titrated with HCl. Fura-2 recording was performed on a system described earlier [71]. Fura-2 ratios were calibrated *in situ* to calcium concentrations based on formula described by Grynkiewicz [72] with K_d_ for Fura-2: 224 nM.

### Reverse transcription PCR

RNA was isolated using RNeasy Mini Kit (Qiangen 74104) following the manufacturer’s instructions. RT-PCR was analysed with QIAGEN OneStep RT-PCR Kit (210212) with amplification parameters as follows: one cycle at 50 °C for 30 min and one cycle at 95 °C for 15 min followed by 37 cycles at 95 °C for 30 s, 58 °C for 30 s, 72 °C for 40 s, and one final cycle at 72 °C for 10 min. The following primers were designed using Primer BLAST and used for TGR5 amplification: human TGR5 forward 3′ TCCTGCCTCCTCGTCTACTT 5′ human TGR5 reverse 3′ GGTAGGGGGCTGGGAAGATA 5′(247 bp), human FXR forward 3′AGAGATGGGAATGTTGGCTGAA 5′ human FXR reverse 3′ GTGAGTTCAGTTTTCTCCCTG 5′(186 bp), rat TGR5 forward 3′ GCTACTGGAGTGGTAGGCAG 5′ rat TGR5 reverse 3′ TCAGTCTTGGCCTATGAGCG 5′(225 bp). All primers were synthesised by TAG Copenhagen A/S (Denmark).

### Western blot

Protein lysates were prepared by adding lysis buffer (50 mM TrisBase, 0.25 M NaCl, 5 mM EDTA, 1 % Triton X-100, and 4 mM NaF) containing protease inhibitor. Cell lysates were centrifuged at 15,000 g for 15 min at 4 °C. To obtain the membrane microdomain enriched samples the lysate was centrifuged at 200,000 g for 1 h (Beckman Ultracentrifuge Ti 70.1 Rotor) [61]. Western blot samples were denatured by heating to 37 °C in 50 mM dithiothreitol for 30 min and run on precast gels from Invitrogen. The membranes were blocked overnight at 4 °C in 0.5 % milk powder and 1 % BSA. Primary antibody for TGR5 (1:400 rabbit, Abcam ab72608) were added in blocking buffer for 1.5 h. The goat anti-rabbit secondary antibody conjugated to horse-radish peroxidase (1:2.500) was added in blocking buffer, for 1 h. EZ-ECL chemiluminescence detection kit for HRP (BI, Biological Industries) was added and blots were viewed on Fusion FX Vilber Lourmat.

### Immunocytochemistry

AR42J cells were grown on glass coverslips (similar as for dishes, see above) and Capan-1 cells were seeded on collagen coated Snapwells. The cells were gently washed with physiological PBS and fixed in 4 % paraformaldehyde in PBS for 15 min, treated with 0.1 M TRIS-glycine (pH 7.4) for 15 min, and then rinsed in PBS and permeabilized for 10 min in PBS with 0.5 % TritonX-100. Cells were blocked with 10 % BSA in PBS for 45 min and then incubated with TGR5 (1:400; Abcam) for 1.5 h. Slides were washed for 10 min and then incubated 1 h with 1:400 goat anti-rabbit secondary antibody conjugated to Alexa 488 (Life Technology). For nuclear staining, DAPI was used (1:400) and mounted with DAKO fluorescent mounting medium. Slides were viewed using a 40X N.A 1.3 objective with TCS SP 5X.

### Statistics

Data are shown as the mean values ± S.E.M. To test the statistical significance between two conditions, unpaired two-tail Student’s *t* test was applied. For multiple conditions, one-way ANOVA with Bonferroni’s Multiple Comparison Test was used. *P* < 0.05 was considered statistically significant. For FLIM-FRET analysis and statistics see above.

## Additional file

Additional file 1: Figure S1. Effect of GPBAR-A on intracellular Ca^2+^ response from Capan-1 cells. **Figure S2.** Effect of (A) CDCA and (B) ATP release inhibitors on standard curves.

## Abbreviations

ATP_i_: Intracellular Adenosine 5′ – triphosphate
ATP_e_: Extracellular Adenosine 5′ – triphosphate
BA(s): Bile acid(s)
CDCA: Chenodeoxycholic acid
GCDCA: Glycochenodeoxycholic acid
CFTR: Cystic fibrosis transmembrane conductance regulator
[Ca^2+^]_i_: Intracellular Ca^2+^ concentration
FLIM: Fluorescence lifetime imaging
FRET: Fluorescence resonance energy transfer
FXR: Farnesoid X receptor
GPBAR-A: 4-[[3,5-Bis(trifluoromethyl)phenyl]methyl]-6-(2-fluorophenyl)-4,5-dihydro-pyrido[3,2-*f*]-1,4-oxazepin-3(2*H*)-one
MgGreen: Magnesium Green indicator
NCX: Sodium/calcium exchanger
NEM: N-Ethylmaleimide
P2R: P2 purinergic receptor
PPADS: Pyridoxal phosphate-6-azo(benzene-2,4-disulfonic acid) tetrasodium salt hydrate
SERCA: Sacro/endoplasmatic reticulum Ca^2+^ ATPases
TCDCA: Taurochenodeoxycholic acid
TGR5: G protein-coupled bile acid receptor
VNUT: Vesicular NUcleotide Transporter
