# Supplementary material for: Bile acid effects are mediated by ATP release and purinergic signalling in exocrine pancreatic cells
Source: Cell Commun Signal. 2015 Jun 9;13:28. doi: 10.1186/s12964-015-0107-9 (PMC4459444; doi:10.1186/s12964-015-0107-9)
Supplement: Additional file 1: Figure S1. — Effect of GPBAR-A on intracellular Ca2+ response from Capan-1 cells. Figure S2. Effect of (A) CDCA and (B) ATP release inhibitors on standard curves. [file 12964_2015_107_MOESM1_ESM.pdf]

Additional figures

Figure S1 Effect of GPBAR-A on intracellular Ca^{2+} response from Capan-1 cells

A

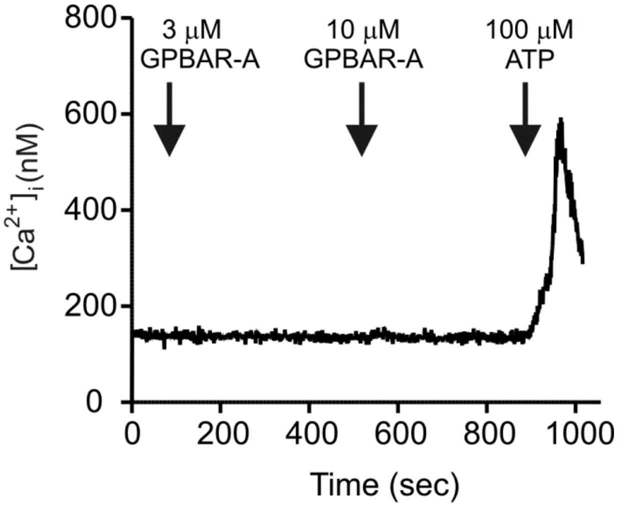

Figure S1 (A) Representative recording of $[Ca^{2+}]_i$ transient in Capan-1 cells after stimulation with GPBAR-A (3 and 10 µM; n = 3) in non - perfused conditions. Addition of GPBAR-A did not increase $[Ca^{2+}]_i$ response in contrast to control ATP (100 µM).

Figure S2 Effect of (A) CDCA and (B) ATP release inhibitors on standard curves

A

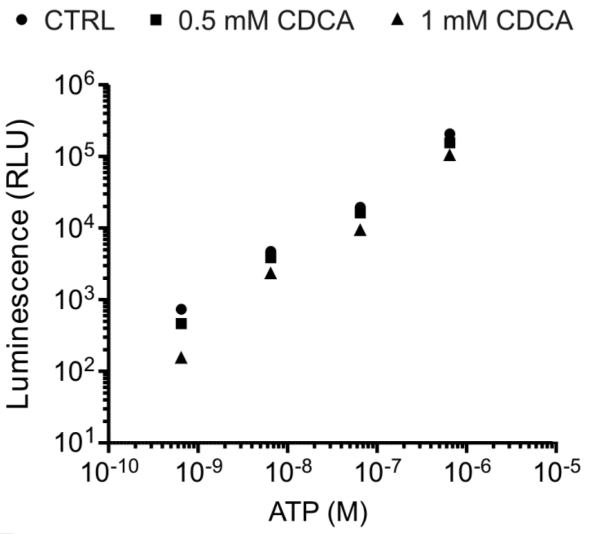

B

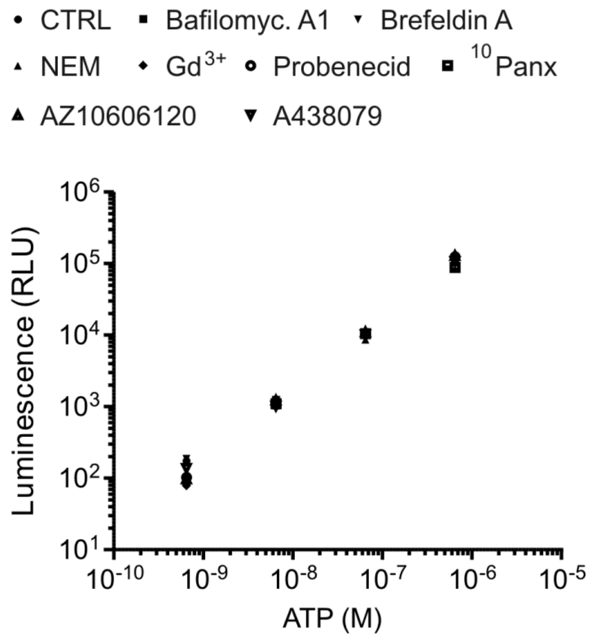

A. Standard curve showing the effect of 0.5 and 1 mM CDCA on luciferin/luciferase activity. **B**. Standard curve presenting the fffect of ATP release inhibitors: Bafilomycin A1 (1 µM), Brefeldin A (5 µg/ml), NEM (N-Ethylmaleimide, 250 µM), Gd3+ (Gadolinium chloride, 50 µM), probenecid (500 µM), pannexin mimetic peptide (100 µM) and P2X7 receptor inhibitors AZ10606120 (10 µM), A438079 (10 µM).
